# Citric Acid: A Multifunctional Pharmaceutical Excipient

**DOI:** 10.3390/pharmaceutics14050972

**Published:** 2022-04-30

**Authors:** Maria Lambros, Thac (Henry) Tran, Qinqin Fei, Mike Nicolaou

**Affiliations:** 1 Department of Pharmaceutical Sciences, College of Pharmacy, Western University of Health Sciences, 309 E Second Street, Pomona, CA 91766, USA; thac.tran@westernu.edu (T.T.); qfei@westernu.edu (Q.F.); 2 Doric Pharma LLC, 5270 California Ave, Suite 300, Irvine, CA 92617, USA; mnicolaou@doricpharma.com

**Keywords:** citric acid, excipient, formulation, citrates, lyophilization, co-crystals, co-amorphous, proteolytic inhibitor, effervescence, taste masking

## Abstract

Citric acid, a tricarboxylic acid, has found wide application in the chemical and pharmaceutical industry due to its biocompatibility, versatility, and green, environmentally friendly chemistry. This review emphasizes the pharmaceutical uses of citric acid as a strategic ingredient in drug formulation while focusing on the impact of its physicochemical properties. The functionality of citric acid is due to its three carboxylic groups and one hydroxyl group. These allow it to be used in many ways, including its ability to be used as a crosslinker to form biodegradable polymers and as a co-former in co-amorphous and co-crystal applications. This paper also analyzes the effect of citric acid in physiological processes and how this effect can be used to enhance the attributes of pharmaceutical preparations, as well as providing a critical discussion on the issues that may arise out of the presence of citric acid in formulations.

## 1. Introduction

The global production of citric acid in 2020 reached 2.39 million tons, and by 2026, citric acid production is projected to increase to 2.91 million tons [1]. The pharmaceutical industry utilizes 12% of the global production, whereas 70% is utilized by the food industry [2]. Citric acid has many applications, including in flavoring, buffering, and as a chelating agent in the food and beverage industry. Many drinks and sweets have an appealing tart taste owing to citric acid. It is also used as a pH regulator [3]. Its effervescence in the presence of carbonates make it useful as tablet disintegrant, in instant drinks, and in personal care products, such as bath tablets [4,5]. Citric acid monohydrate is the usual form of citric acid sold on the commercial market. It is produced via crystallization from cold, saturated solutions through slow evaporation. Citric acid anhydrous is produced from hot, saturated solutions of citric acid [6].

**Figure 1:**
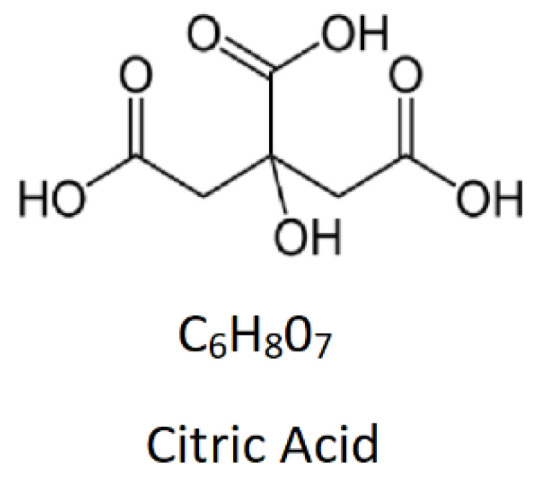
Chemical structure and molecular formula of citric acid.

Citric acid is a weak tricarboxylic acid found in citrus fruits like lemons, which contain 7–9% citric acid according to their dry weight. The three carboxylate groups of citric acid monohydrate have different pKa values, namely 3.15, 4.78, and 6.40 [7]. Until 1919, lemons were the main source of citric acid (Figure 1). Afterwards, the fungus *Aspergillus niger* was used for the production of citric acid at commercial scale [8]. Since then, other *Aspergillus* species such as *A. flavus*, *A. awamori*, *A. nidulans*, and *A. wentii* have been used to produce citric acid in the presence of sugars; however, *A. niger* remains the main source of citric acid production. Yeasts can also be used to produce citric acid from carbohydrates and n-alkanes. Yeasts used for such a purpose include the genera *Candida*, *Saccharomyces*, *Zygosaccharomyces*, *Kloeckera*, *Torulopsis*, *Debaryomyces*, *Pichia*, *Torula*, *Yarrowia*, and *Hansenula* [8,9,10].

The tribasic salt of citric acid, trisodium citrate, is commercially available in both anhydrous and dihydrate forms. The anhydrous form dissolves faster than its dihydrate form. Furthermore, the anhydrous form of trisodium citrate is porous and has good flowability, even in the presence of moisture, due to its ability to take up water or other fluids. It is also used as a carrier for liquids while retaining its flowability and compressibility [11]. Detailed analyses of the structural, spectral, and thermal properties of anhydrous citric acid have been reported [12].

## 2. Citric Acid and Physiological Considerations

### 2.1. Citric Acid Transporters

**Figure 2:**
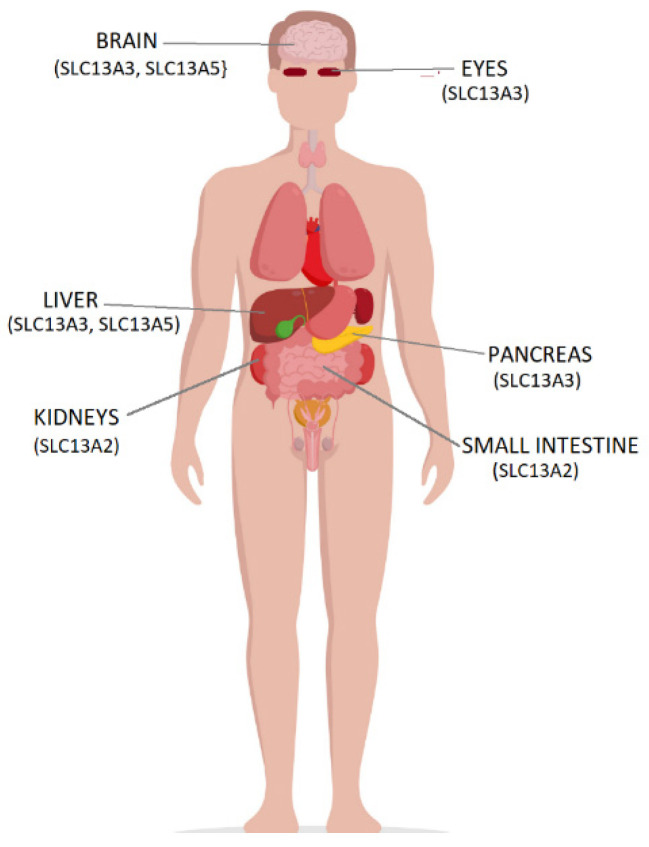
The different citrate transporters and their locations in the body.

Citric acid, along with its metabolic intermediates of the Krebs cycle (citric acid cycle) such as citrates, succinates, and alpha-ketoglutarate, is transported by certain members of the solute carrier family 13 (SLC13) transporters, namely, SLC13A2 or Na^+^/dicarboxylate cotransporter 1, NaDC1; SLC13A3 or Na^+^/dicarboxylate cotransporter 3, NaDC3; and SLC13A5, Na^+^/citrate cotransporter, NaCT, (Figure 2) [13,14,15,16]. Citrates are metabolized mainly in the liver [17].

SLC13A2 is expressed in epithelial tissues with high metabolic needs, such as those in kidneys and small intestine. It takes up protonated citrates in the form of citrates^2−^ and succinates into the cell and plays an important role in the transport of citrates in urine [15,18]. The citrates in urine bind calcium, forming soluble complexes and reducing Ca^2+^ supersaturation of urine; therefore, the presence of citrates in the urine prevents the formation of kidney stones by forming complexes with Ca^2+^ ions and inhibiting crystal formation and aggregation [13].

The SLC13A3 transporter cotransports Na^+^ and dicarboxylate or tricarboxylate ions in a pH-dependent manner. The tricarboxylate transport activity of SLC13A3 is optimal at pH 5.5–6.5 [19]. This transporter is found in the kidneys, specifically on the basolateral membranes of the renal proximal tubules as well as in other tissues such as brain, pancreas, liver, and eye [18]. 

The SLC13A5 or sodium-coupled citrate transporter (NaCT) is selective for tricarboxylate citrate at pH 7.4. It is located in the liver and brain. It is a regulator of metabolic function, energy production, glycolysis, and lipid synthesis [20]. Mutations in the SLC13A5 transporter affect citrate binding and transport and cause epileptic encephalopathy with seizures [21]. Increased expression of the *SLC13A5* gene is linked to type 2 diabetes, gluconeogenesis, and non-alcoholic fatty liver disease. *SLC13A5* is the mammalian homolog of the *Indy* (I am not dead yet) gene, which is highly expressed in the liver. Reduced expression of the *Indy* gene in lower organisms is associated with longevity [22]. The deletion of *SLC13A5* in mice mimics caloric restriction, without reducing the intake of calories and protects mice from hepatic fat accumulation and aging-induced obesity [23]. 

### 2.2. Citrates and Tight Junctions/Absorption

Citrates chelate calcium resulting in disruption of tight junction integrity. Indeed, calcium chelation is an effective way to loosen up tight junctions and increase paracellular absorption [24]. This was shown in a previous study using ruthenium red, an electron-dense compound that does not permeate cells and is used as a marker of tight junction integrity. The transcellular electrical resistance of the duodenum and jejunum was reduced in the presence of sodium citrate. Goblet cells, which have tight junctions, were surrounded by abundant ruthenium red deposition in the presence of citrates [25]. Calcium from milk mixed with fruit juice containing citrates was shown to have higher uptake and transport through Caco 2 cells compared to calcium from baby formula, which lacks citrate [26]. Furthermore, citric acid enhances the calcium availability as is shown by an in vitro simulated gastrointestinal digestion method [27]. In the presence of citric acid, the intestinal pH is reduced, while the absorption of calcitonin is increased [28]. Because citrates increase GI absorption through their interference with tight junctions, they may also increase the absorption of other ions, such as lead and aluminum. Citrate-enhanced GI absorption of lead and aluminum has been shown in numerous studies [29,30]. Dietary citric acid from lemon juice, when consumed together with Al(OH)_3_, increases the absorption of aluminum, as shown by high amounts of Al^3+^ in the bones or blood or by aluminum excretion in urine. This may result in acute aluminum toxicity, which is especially undesirable in the case of patients who suffer from chronic kidney dysfunction. Extensive aluminum deposition in bones has been found in patients who receive citrate in combination with Al(OH)_3_ [30,31].

The ability of citric acid to remove inorganic matter via complexation makes its use popular in dentistry. Solutions containing 5–50% citric acid are used to irrigate the root canal and to remove inorganic components of the root dentine surface of the canal wall and increase its permeability in order for the filling materials to adapt to the root canal and increase the bond strength of resin endodontic sealers to root dentine [32,33,34].

### 2.3. Proteolytic Inhibitor

The absorption of protein drugs is hindered not only by absorption barriers such as the intestinal wall but also by the action of proteolytic enzymes that degrade proteins and reduce their concentration, thereby reducing absorption. Reducing the activity of these proteolytic enzymes (chymotrypsin, trypsin) enhances the absorption of protein drugs; this can be achieved by reducing the pH in the local microenvironment of the enzyme or by reducing the concentration of calcium ions [35].

Citric acid is used in oral protein and peptide formulations as it inhibits proteolysis. For example, the enteric-coated tablet TBRIA™, which contains citric acid, is an oral formulation of salmon calcitonin used in the treatment of postmenopausal osteoporosis. After the degradation of the enteric coating in the duodenum, the protein or peptide active pharmaceutical ingredients (APIs) are released along with the citric acid. The presence of citric acid keeps the pH low enough to inhibit the activity of proteolytic enzymes so that they cannot degrade the protein or peptide APIs, facilitating better absorption [24,36,37]. Citric acid has also been used to avoid the proteolysis of salmon calcitonin in a mini-sphere emulsion-based formulation [38]. 

**Figure 3:**
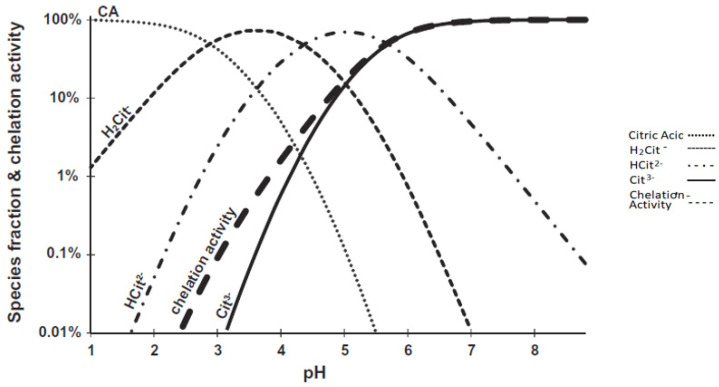
Percent fractions of various citric acid (CA) species and their chelation activity calculated at different pH values. The chelation activity (boldly dashed line) is at its maximum above pH 7, when the Cit ^3−^ concentration is also at its highest. Reprinted with permission from Ref. [37] Copyright 2014, Elsevier.

Citrates may impair the activity of proteolytic enzymes via calcium chelation. Both citrates and EDTA chelate Ca^2+^ and reduce the activity of chymotrypsin. The proteolysis of insulin, which is mainly due to chymotrypsin, is significantly reduced in the presence of citrates at pH > 7, as calcium complexes with citrates in this pH range resulting in the reduction of chymotrypsin activity. While citric acid acts as a calcium ion chelator at pH > 7, at pH 3–4 range, citric acid exerts inhibitory action against proteolytic enzymes acting as an acidifier [37]. Figure 3 shows the calculated concentration of different forms of citric acid such as H_2_Cit^−^, HCit^2−^, and Cit^3−^, and the chelation activity at various pH levels [37]. Citrate chelating activity for divalent cations is maintained even after interaction of citrates with chitosan chains; chitosan citrate at pH 6.5 enhances the penetration of peptidic drugs and shows strong inhibition of zinc dependent peptidases such as carboxypeptidase A and leucine aminopeptidase [39].

### 2.4. Bone and Citrates

Citrate is an important part of bone structure. Eighty percent of the body’s total citrate content is found in the bones. It is strongly bound to the surfaces of the apatite nanocrystals and stabilizes them [40]. Citrates also play an important role in new bone regeneration. It has been shown that citrate is secreted by osteoblasts and is incorporated into bone during bone formation [41,42]. Citrates regulate apatite nanocrystal growth, affecting bone strength, stability, and fracture resistance [43], via incorporation between mineral platelets forming citrate bridges between the platelets [44]. Citric acid used in superficial demineralization of tooth root surfaces enhances the proliferation and spreading of osteoblasts and the regeneration of cementum [45,46]. 

Citric acid has been used in biomimetic materials as a scaffold for osteogeneration via deposition of hydroxyapatite [47]. The presence of citrate, either in the cell culture media or in the polymer itself, supports osteoblast proliferation and differentiation, and enhances the deposition of apatite, thereby enhancing the strength of the material [42,48]. Crosslinked, urethane-doped octanediol citrate polymers show enhanced hydroxyapatite binding, while citric acid supplementation promotes osteoblast culture [49]. Citric acid-based hydroxy-apatite materials form strong biomimetic and biocompatible composite scaffolds that have been produced employing click chemistry and can be used as osteogenic implants to repair orthopedic defects [50]. 

## 3. Citric Acid in Formulations

### 3.1. Citric Acid in Taste-Masking and Effervescence

The effectiveness of a therapeutic treatment depends on patient compliance. Taste acceptance by the patient enhances compliance. This is especially important with orally disintegrating tablets (ODTs), as the drug is released in the mouth [51]. Such ODTs are used to improve the oral delivery of therapeutics in children, in elderly patients with dysphagia, and in patients with epileptic seizures [52,53]. Several of these drugs have an unpleasant or bitter taste, making patient compliance difficult, and taste-masking is important in such cases. 

Citric acid is used to mask the bitter taste of drugs and improve their palatability. In fact, the presence of citric acid is positively correlated with acidity, sour taste, citrus aroma and flavor, and is negatively correlated with bitterness [54]. For example, epinephrine has a bitter taste, and while the addition of two artificial sweeteners, aspartame and potassium acesulfame, can reduce the bitterness to an acceptable level, bitterness was only reduced to an undetectable level following the addition of citric acid into these formulations [55]. Similarly, the addition of citric acid along with sweeteners reduced the bitter taste of olopatadine, mirtazapine, and diclofenac [55,56,57,58]. Additionally, concentration of 1% citric acid was used to mask the bitterness of famotidine in microspheres incorporated into orally disintegrating tablets [59]. 

Effervescent formulations that contain citric acid and sodium bicarbonate can achieve a pleasant mouthfeel sensation on the tongue and in the mouth [60]. It has been shown that the unpleasant taste of functionalized calcium carbonate and calcium phosphate was masked in effervescent formulations containing citric acid [60]. Additionally, citric acid can be used to stimulate salivary glands and is considered as one of the most effective stimulants to induce high salivary flow [61,62]. The ability of citric acid to mask taste has been evaluated using human volunteers and e-tongues [63]. 

Effervescence is commonly used in orally administered pharmaceutical formulations. The term “effervesce” refers to the release of gas when an acid and base interact with water. Usually, the acid is citric acid, and the base is sodium bicarbonate or sodium carbonate [64]. While several other acids can be used in place of citric acid, such as malic, fumaric, tartaric, and adipic acids, citric acid is most widely used in effervescence formulations because it imparts a pleasant citrus-type flavor and acts as a flavor enhancer [65]. Effervescent tablets or powders in the presence of water or another liquid, such as saliva, release gas as carbon dioxide (CO_2_) and can be used to produce carbonated liquid drinks. 

Since effervescence can quickly disperse active compounds and allows rapid dissolution, it is used in the administration of pharmaceuticals, particularly in patients who face difficulty in swallowing a tablet or capsule [66]. Another advantage of effervescence is that it also allows quick dispersion of the active compounds in the oral cavity as well as absorption through oral mucosal and, in this way, the first-pass effect can be avoided, leading to increased bioavailability of the drug and faster activity onset [5,67]. Effervescence may increase buccal absorption since the citric acid in the formulation sequesters calcium ions (Ca^2+^), making the tight junctions more permeable, thereby promoting paracellular transport [35]. 

An effervescent mixture may contain citric acid and sodium bicarbonate, or carbonate. The molar ratio of citric acid to bicarbonates is 1:3 as is shown below (1). When carbonates are used instead of bicarbonates, the molar ratio of citric acid to carbonates can be 2:3 (2). Effervescence has been used to enhance the solubility of poorly soluble drugs such as atorvastatin, cefuroxime, ketoconazole, metronidazole [68], buspirone [69], fentanyl citrate [70], and bismuth subcitrate [71].

(1) $${\mathrm{CitrH}}_{3}\;+\;3{\mathrm{NaHCO}}_{3}\;\overset{{\;\mathrm{in}\;H}_{2}O}{\to }\;{\;\mathrm{Na}}_{3}\mathrm{Citr}\;\;+\;3{\mathrm{CO}}_{2}↑\;+\;3H_{2}O\;\;\mathrm{Citric}\;\mathrm{Acid}\;\;\mathrm{Sodium}\;\mathrm{Bicarbonate}\;\;\mathrm{Trisodium}\;\mathrm{Citrate}\;\;\mathrm{Carbon}\;\mathrm{Dioxide}\;\;\mathrm{Water}$$

(2) $$2{\mathrm{CitrH}}_{3}\;+\;3{\mathrm{Na}}_{2}{\mathrm{CO}}_{3}\;\overset{{\;\mathrm{in}\;H}_{2}O}{\to }\;2{\mathrm{Na}}_{3}\mathrm{Citr}\;\;+\;3{\mathrm{CO}}_{2}↑\;+\;3H_{2}O\;\;\mathrm{Citric}\;\mathrm{Acid}\;\;\mathrm{Sodium}\;\mathrm{Carbonate}\;\;\mathrm{Trisodium}\;\mathrm{Citrate}\;\;\mathrm{Carbon}\;\mathrm{Dioxide}\;\;\mathrm{Water}$$

The effervescence reaction of citric acid with bicarbonates, which releases CO_2_, is also used to develop tablets that float in the stomach. The CO_2_ gas produced during the effervescent reaction is trapped within the gel polymers of the tablet, initiating buoyancy. The buoyant tablets float in the gastric juice of the stomach for a longer time compared to regular tablets, releasing the drug to be absorbed by the stomach over longer periods, thus increasing its bioavailability [72]. This technology is used for drugs that are unstable or not soluble at intestinal pH [73], and has been expanded for used in extended-release floating granules [74]. Effervescent, gastro-retentive floating tablets of verapamil [75], calcium disodium edentate [76], ciprofloxacin [77], dipyridamole [73], lisinopril [78], and venlafaxine [79] are examples of this technology.

### 3.2. Citric Acid in the Lyophilization Process

Citric acid is a common excipient in lyophilized formulations [80]. Lyophilization or freeze-drying is an important process of pharmaceutical significance and is used in the preservation of labile pharmaceuticals. Lyophilization involves removal of frozen water from a formulation via sublimation to produce a powder that can be reconstituted before use. The lyophilized products are amorphous, solid-state, glassy materials that are considered to be supercooled liquids. Important parameters in the lyophilization process include buffering, for pH control, and the glass transition temperature, Tg. Components of the buffer during the freeze-drying process may crystallize and affect the pH of the solution. When buffer components of the solution crystallize at subzero temperatures, phase separations, solid phases, and liquid phases are created within the solution, which have different pH values depending on the freezing rate and components [81]. Phase separation during the lyophilization process must be avoided as it may lead to pH changes and degradation of the formulation components.

Citrate buffers are used extensively in lyophilized products because they usually do not crystallize during the process and remain amorphous, with minimal pH changes, unlike sodium phosphate buffers, which are known to crystallize during lyophilization [82]. Mannitol is an important lyoprotectant for peptides and proteins and should remain amorphous and not crystallize during lyophilization [83,84]. The presence of 1% or 5% of sodium citrate in mannitol solutions has been shown to inhibit mannitol crystallization during lyophilization [85]. The crystallization process is also pH dependent. Citric acid solutions are crystallized at pH 4 and not at pH 5 or 6 [86]. It has been also reported that 1 M citric solutions exhibit long-lived, large-scale inhomogeneities, i.e., supramolecular structures [87]. 

During lyophilization, sucrose in the presence of citric or other acids inverts (converted into fructose and glucose), even at low temperatures and with very low amounts of water (less than 0.1%); for this reason, care must be exercised. The inversion of sugar must be avoided as the products from sugar inversion may react with other components of the formulation and cause decomposition. Although sucrose inversion depends on the pH of the solution, it may also happen after lyophilization. This is due to citric acid, even in the solid state, retaining its degree of ionization and possibly causing sucrose protonation, which in turn results in sucrose inversion [88]. 

The glass transition temperature, Tg, of anhydrous citric acid is 11 °C, whereas the Tg of citric acid monohydrate (that is, in the presence of an equimolar amount of water or 8.6% water content) is −25 °C. The glass transition temperature of the maximally freeze-concentrated solution, Tg′, of citric acid is −53 °C [80,89]. The low Tg and Tg′ of hydrated citric acid explains the difficulty in keeping pure citric acid from crystallizing when in amorphous state [80]. Citric acid shows significant changes in its viscosity in the vicinity of the Tg, with non-Arrhenius behavior over a broad range of temperatures, as is observed with other small molecules [89].

Using a system of trehalose–citrate and sulfonephthalein with a pH indicator as a probe, it has been shown that at a certain pH, the protonation of sulfonephthalein was higher in the lyophilized state compared to its protonation in solution before the lyophilization [90].

### 3.3. Citric Acid in Polymers

The three carboxylic groups and one hydroxyl group of citric acid allow it to react and crosslink with other biocompatible multifunctional materials such as glycerol, cellulose, and sebacic acid via condensation reactions, forming crosslinked ester copolymers capable of drug delivery. The advantages of condensation reaction include that it is considered “green”, is catalyst-free, and enables ester bond formation. 

Reacting citric acid with glycerol under a range of temperatures (90–150 °C) results in the formation of biodegradable ester copolymers. Incorporating the antibiotic gentamycin into this polymer resulted in effective bacterial killing [91]. The kinetics of citric acid and glycerol polycondensation at three different temperatures was recently studied [92]. The generation of citric acid–glycerol copolymers in the presence of benzene and p-toluenesulfonic acid (PTSA) has also been reported [93]; however, this type of synthesis raises concerns regarding biocompatibility because both benzene and PTSA are known carcinogens [91]. Biocompatible poly(diol citrate) elastomers were formed by reacting citric acid with different type of diols. The mechanical properties, such as the stiffness and degradation characteristics, can be controlled through the choice of the diol used and the crosslinking density [94]. The polyfunctionality of citric acid, its low cost, and its ability to react in polycondensation reactions with other nontoxic materials without requiring catalysts make it an important component used for the formation of biomaterials in regenerative engineering. An insightful review of design considerations and uses of citric-acid-based polymeric biomaterials in regenerative engineering has been published [49]. 

Hydrogels consisting of crosslinked polymers derived from citric acid, polyethylene glycol 200, and maleic acid can be used for drug delivery purposes [95]. Owning to its multifunctionality, citric acid can form dendrimers with polyethylene glycol that can trap small drug molecules such as mefenamic acid, diclofenac, and naproxen. The trapped small molecules are released in a controlled manner for up to seven hours [96,97]. 

**Figure 4:**
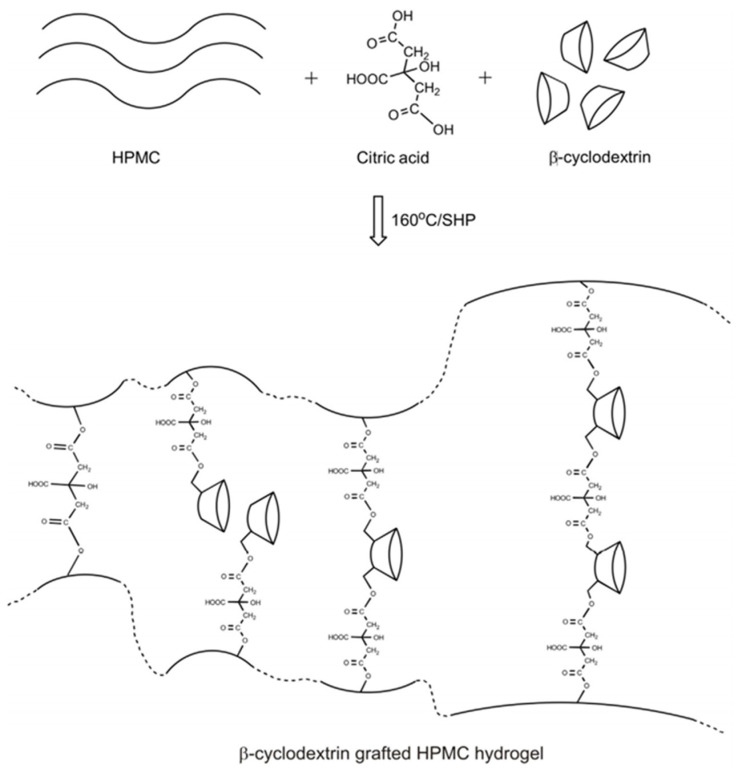
Citric acid, with its 3 carboxyl groups and one hydroxyl group, can serve as a crosslinker between polymer molecules and as a linker to link cyclodextrins and a polymer, such as HPMC. Reprinted with permission from Ref. [99]. Copyright 2016 Elsevier.

Crosslinking of citric acid with cyclodextrins (CDs) produces polymers that can enhance the solubility of poorly soluble drugs compared to cyclodextrin monomers [98]. Cyclodextrins are water-soluble cyclic polysaccharides. They have a cone shape, and the external surface of the cone is hydrophilic, while the internal surface, which is the cavity of the cone, is hydrophobic. Lipophilic drugs partition into the cyclodextrin cavity. The formation of drug–cyclodextrin complexes enhances the solubility and bioavailability of drugs that are difficult to solubilize. The three main types of cyclodextrins (CDs), namely alpha-CDs, beta-CDs, and gamma-CDs, have different cavity sizes, with gamma-CDs having the largest cavity and broadest solubility. Citric acid is used as a crosslinking agent to graft beta-cyclodextrins to hydroxypropylmethylcellulose (HPMC) hydrogel films for ketoconazole delivery (Figure 4) [99]. The citric acid–cyclodextrin polymers formed through a polycondensation reaction between cyclodextrin and citric acid are non-toxic and environmentally safe. Citric acid–CD polymers enhance the solubility of drugs, such as albendazole [100], ciprofloxacin [101], ethoxzolamide [102], doxorubicin [103], bupivacaine, risperidone, paliperidone, and promethazine [104]. Citric acid has been used to develop coatings for biodegradable cardiovascular stents. Specifically, the biodegradable alloy of Mg-Zn-Y-Nd was coated with successive layers of polydopamine, citric acid, and Arg-Gly-Asp (RGD) peptide. The surface that was created is hemocompatible and allows for endothelization, while inhibiting smooth muscle cell adhesion and proliferation, overall improving the biocompatibility of the Mg alloy stents [105,106]. Extensive reviews of the role of citric acid as a crosslinker can be found in references [107,108].

### 3.4. Co-Amorphous Drugs and Co-Crystals

#### 3.4.1. Co-Amorphous Drugs

When comparing the solubility and dissolution rate of the crystalline versus amorphous form of a drug, the amorphous form is more soluble and will have a faster dissolution rate. The amorphous form, however, may not be thermodynamically stable and will revert, in time, to the crystalline form. To overcome this instability, initial efforts are focused on mixing the drug substance with a polymer, whereby polymer chains act as a barrier, separating the drug molecules from each other. Additionally, intermolecular interactions between the polymer and the drug contribute to providing a stable amorphous solid dispersion (ASD) mixture. The disadvantages of these ASDs are that: (a) the polymers represent a high percentage of the drug–polymer mixture, requiring a larger tablet (or more tablets) to deliver the required drug dose, resulting in dosage form burden [109]; (b) the hydroscopic tendencies of the polymer may result in moisture absorption, a lower glass transition temperature, Tg, and increased molecular motility, resulting in phase separation between the polymer and the drug and eventual re-crystallization of the drug [109,110]. 

**Figure 5:**
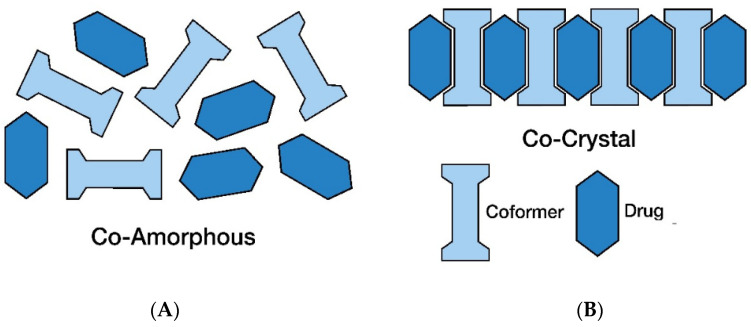
A schematic of co-amorphous (**A**) and co-crystals (**B**).

An alternative solution to avoid the drawbacks of polymer–drug ASDs is to use co-amorphous formulations (Figure 5A). These are combinations of low molecular weight compounds, such as a low MW drug with another drug or with a low MW excipient. For the drug–excipient category, the excipient, i.e., the co-former, is a low molecular weight substance such as an amino acid or carboxylic acid (e.g., citric acid). The co-amorphous formulations between a drug and citric acid can achieve higher drug solubility and dissolution rates compared to the drug alone in its crystalline form [111]. Citric acid, having hydrogen bonding ability due to the three carboxylic groups and one hydroxyl group and a low molecular weight, is useful as a co-former to form co-amorphous structures [112]. The co-amorphous system of ketoconazole–citric acid showed exceptional stability due to its decreased molecular mobility and the presence of structural factors (the three carboxylic groups and one hydroxyl group, which provide opportunities for hydrogen bonds between ketoconazole and citric acid) [113]. Co-amorphous formulations of acyclovir and citric acid formulated in a PEG ointment resulted in enhanced acyclovir penetration compared to crystalline acyclovir [114,115]. Mixtures of paracetamol and citric acid at a 50:50 ratio formed an amorphous blend, having strong hydrogen bond interactions between paracetamol and citric acid that were stable under dry conditions for at least 27 weeks [116].

A loratadine–citric acid co-amorphous system prepared using the solvent evaporation technique had enhanced physical stability [117]. Citric acid and sulfathiazole co-milled together formed a co-amorphous preparation that was stable up to 28 days at RH 10% [118]. At higher RH, the co-amorphous sulfathiazole crystallized to different polymorphs. Additionally, separation of the citric acid from the co-amorphous indomethacin–citric acid preparations containing more the 30% citric acid was observed [119]. Sometimes, in order to enhance stability, instead of using citric acid alone as a co-former, citric acid can be used as a part of the co-former as was the case for citric acid–L-arginine, which was used as a co-former for carbamazepine [120]. Citric acid can interact with basic drugs and form hydrogen bonds leading to drug amorphization and increased solubility [121]. The solubility of difficult to solubilize basic drugs such as haloperidol and itraconazole was increased when these drugs interacted with citric acid and formed stable amorphous solid dispersions [122,123].

#### 3.4.2. Co-Crystals

The co-formers that form co-amorphous structures, under certain preparation, time, relative humidity (RH), and temperature conditions, can also form co-crystals. In co-crystals, the co-formers can interact stoichiometrically and form a 3D ordered structure that leads to a crystal lattice (Figure 5B). Co-crystals can be formed by any pair of electron donors and acceptors, whereas crystalline salts of weak acids or bases require proton transfer between the components [124]. Co-crystals represent a homogeneous phase of one type of a crystal lattice and are not mixtures of two different phases of pure crystalline components [125]. An important distinction between co-crystals and solvates is that co-crystals are made of an API and a co-former, which in their pure state are solid under ambient conditions, whereas solvates contain a solvent or water as a guest molecule [126,127,128]. Co-crystals are of interest in the pharmaceutical industry because an API in co-crystal form has a better dissolution rate than in its pure crystal form or other forms, such as salts, hydrates, or polymorphs [129,130]. 

**Figure 6:**
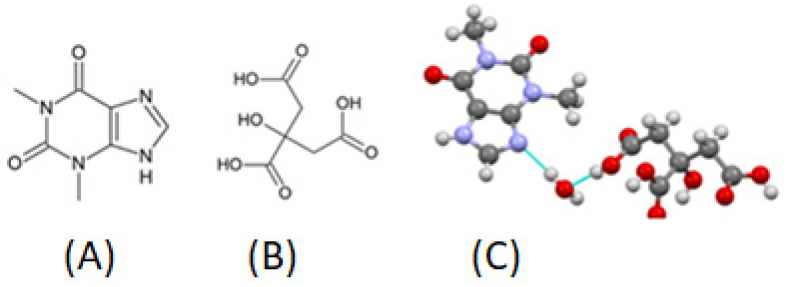
Theophylline (**A**) and citric acid (**B**) through resonant acoustic wet granulation form a co-crystal (**C**). Reprinted with permission for Ref. [134]. Copyright 2021 MDPI. This image is licensed under CC BY 4.0.

When citric acid interacts with APIs to form co-crystals, citric acid is the co-former that combines with the API through hydrogen bonds, π-stacking, and van der Waals forces to form these co-crystals [126,131]. Physical mixtures of theophylline and citric acid at 1:1 stochiometric ratio when stored at 55 °C and 75% RH for 24 h are transformed into co-crystals [125,132]. Grinding theophylline and citric acid together for 60 min or using slow solvent evaporation can also be used to form co-crystals [133]. Acoustic resonant granulation is a new efficient technique that resulted in complete theophylline–citric acid co-crystal formation (Figure 6), as well as simultaneous granule formation, whereas high-shear wet granulation resulted in a low co-crystal yield [134]. Mixtures of paracetamol and citric acid, through slow solvent evaporation, formed co-crystals at a 2:1 paracetamol-to-citric acid stochiometric molar ratio [131]. 

As discussed above, theophylline, and other xanthines such as caffeine can form co-crystals with citric acid and the presence of water assists in this formation. Table 1 lists different API–citric acid co-crystals, their stochiometric ratio, methods of preparation and techniques used for their study. Caffeine–citric acid co-crystals at a 1:1 stoichiometric ratio can be formed using liquid (water)-assisted grinding [135,136]. Caffeine–citric acid co-crystals show two polymorphs [137]. Cafcit^®^ caffeine citrate, a marketed product for infantile apnea, is formulated using caffeine–citric acid co-crystals [138]. Anhydrous theophylline–citric acid co-crystals are prepared in ethanol, while theophylline–citric acid co-crystal hydrates (1:1:1), which are prepared in water, contain three types of molecules in the crystal, namely theophylline, citric acid, and water. Since citric acid as a co-former is water-soluble and takes up moisture, its co-crystals are prone to conversion. Theophylline–citric acid co-crystal hydrates can convert to and from anhydrous theophylline–citric acid co-crystal, depending on the relative humidity or excipients that affect the water activity [139]. 

### 3.5. Citric Acid in Formulations, Injection Pain and Other Issues

Citric acid has wide use in formulations, including in parenteral administration dosage forms, such as intramuscular or subcutaneous injections. Injections that contain an acid are painful [151,152]. There are several papers in the literature that attribute injection pain to the presence of citric acid or a citric or citrate buffer. Comparing the injection pain caused by different buffers, the formulation with a citrate buffer caused more pain [153]. For example, adalimumab (Humira^®^), which is administered through subcutaneous injection for the treatment of rheumatoid arthritis, ulcerative colitis, and Crohn’s disease, also comes in a citrate-free version [151,154].

Citric acid activates the acid-sensing ion channel 1 (ASIC1), while neutral citrate does not. The latter chelates calcium ions that act as inhibitors to the ASIC1 [151]. Citric acid also acts on TDAG8 coupled with TRPV1 to induce itching-like behavior in mice [152]. In general, protons are the major culprits involved in injection pain. Injection pain is unpleasant and may contribute to patient non-compliance, leading patients to skip doses or stop therapy altogether, with detrimental effects on their therapeutic outcomes. To avoid injection pain, clinicians add a small amount of bicarbonate into the injection just before administration. Also, eliminating citrates from the formulation may avoid injection pain. However, since citric acid chelates extracellular Ca^2+^ that normally inhibits ASIC1, adding inhibitors of ASIC1 or adding supplemental Ca^2+^ may also help in avoiding injection pain, without the need to eliminate citrates from the formulation [151]. Better understanding the mechanism of pain caused by citric acid will have important positive therapeutic implications.

Humidity plays an important role in formulation. Crystalline solids at certain relative humidity (RH) turn to solution or deliquesce [155,156]. When RH rises and falls it will cause deliquescence and efflorescence (crystallization), respectively, leading to instabilities. Anhydrous crystalline citric acid at 25 °C and 75% RH turns from solid to solution, deliquescence, whereas citric acid monohydrate is more resistant to humidity and it deliquesces at a higher RH, 78%, at the same temperature [155]. Blends of citric acid with deliquescent solid components result in a lower deliquescent point than deliquescent points of the individual components. For example, the deliquescent points of sucrose and citric acid are 85% and 75% RH, respectively at 25 °C [157], whereas, at the same temperature blends of sucrose and citric acid, have a lower deliquescent point, 64% RH. This may result in chemical (i.e., sucrose inversion) and physical (i.e., caking) instabilities [158]. 

In the case of co-crystals, when the relative humidity is high enough, 98% RH at RT, citric acid, a highly soluble co-former, predisposes the co-crystal to convert between anhydrous and hydrated forms, to deliquesce, or to dissociate, in which case the individual components form hydrates [124]. This was the case with theophylline–citric acid where the anhydrous co-crystal was converted to hydrate, as well as with caffeine–citric acid co-crystals where the co-crystal deliquesced and formed a caffeine hydrate, at 98% RH [124,135,139]. Furthermore, impurities at trace level or other excipients such as fructose and xylitol in this type of co-crystal decrease the deliquescent point and affect its stability [139,159].

## 4. Conclusions

Citric acid is an important excipient in many types of pharmaceutical formulations. Its three carboxylic groups and one hydroxyl group provide the functionality and versatility required for its many applications. In this review, we have discussed the fundamental strategies that take advantage of its functionality and have summarized its uses in pharmaceutical formulations. The presence of citric acid in formulations solves many problems but also causes some challenges. Understanding the physicochemical interactions of citric acid with the active pharmaceutical ingredients and other excipients in formulations will promote rational formulation design and effective therapeutics.

## Figures and Tables

**Table 1:** Co-crystals of different APIs and citric acid, their stochiometric ratio, and methods of preparation and study.

Co-Crystal	Stochiometric Ratio	Methods of Preparation	Techniques Used to Study	References
Berberine Chloride–Citric Acid	1:1	Grinding	X-ray, FTIR, DSC, Dynamic Water Vapor Sorption (DVS) HPLC, Dissolution	[140]
Dapagliflozin–Citric Acid	1:1	Evaporation	X-ray, FTIR, DSC, TGA, ^1^HNMR, HPLC, SEM, DVS, dissolution	[141]
Nefiracetam–Citric Acid	2:1	Slow Evaporation	X-ray, DSC, TGA, DVS, HPLC UV	[142]
Nitrofurantoin–Citric Acid	1:1	Liquid-Assisted Grinding Slow Evaporation	X-ray, DSC, TGA, Raman, IR, NMR Elemental Analysis DSC, reflectance FTIR, Polarized Light Microscopy	[143,144]
Piracetam–Citric Acid	Not Reported	Dry Grinding Liquid-Assisted Grinding	X-ray, DSC, FT-Raman	[145]
Praziquantel–Citric Acid	1:1	Liquid-Assisted Grinding	DSC, X-ray, IR	[146]
Theophylline–Citric Acid	1:1	Slow Evaporation Dry Grinding Liquid-Assisted Grinding	DSCFTIR, , Stability Studies	[132,133]
Paracetamol–Citric Acid	2:1	Slow Evaporation	X-ray, DSC, Raman Spectroscopy	[131]
Caffeine–Citric Acid	1:1	Liquid-Assisted Grinding	X-ray, DSC, FTIR	[136,147]
Agomelatine–Citric Acid	1:1	Cooling Crystallization	Ternary Phase Diagrams, Solubility, DITA (Discontinuous Isoperibolic Thermal Analysis)	[148,149]
Creatine–Citric Acid	1:1	Co-milling in Humid Air	X-Ray, TGA, DSC, NMR	[150]

## Data Availability

Not applicable.
